# Planning for a Nondriving Future: Behaviors and Beliefs among Middle-Aged and Older Drivers

**DOI:** 10.3390/geriatrics3020019

**Published:** 2018-04-13

**Authors:** Annie C. Harmon, Ganesh M. Babulal, Jonathon M. Vivoda, Brian J. Zikmund-Fisher, David B. Carr

**Affiliations:** 1 Division of Geriatrics and Nutritional Science, Washington University School of Medicine in St. Louis, St. Louis, MO 63110, USA; dcarr@wustl.edu; 2 Department of Neurology, Washington University School of Medicine in St. Louis, St. Louis, MO 63110, USA; babulalg@wustl.edu; 3 Knight Alzheimer’s Disease Research Center (ADRC), St. Louis, MO 63110, USA; 4 Department of Sociology and Gerontology, Miami University, Oxford, OH 45056, USA; vivodajm@miamioh.edu; 5 Department of Health Behavior & Health Education, University of Michigan School of Public Health, Ann Arbor, MI 48109, USA; bzikmund@umich.edu; 6 Department of Internal Medicine, University of Michigan Medical School, Ann Arbor, MI 48109, USA

**Keywords:** aging drivers, driving cessation, mobility planning

## Abstract

Despite the reality of older adults living many years after driving cessation, few prepare for the eventuality; empirically, planning for a nondriving future has not been directly quantified or explored. The following study quantifies (1) the extent of current drivers’ planning; (2) specific planning behaviors; (3) beliefs about benefits of planning; (4) drivers’ intention to plan more for future transportation needs; and (5) group differences associated with planning. In a predominantly female, black, urban sample of current drivers ages 53–92, fewer than half (42.1%) had planned at all for a nondriving future, with correspondingly low levels of planning behaviors reported. However, over 80% believed planning would help them meet their needs post-cessation and transition emotionally to being a nondriver. Most (85%) intended to plan more in the future as well, indicating further potential openness to the topic. Drivers who planned were older, drove less frequently, limited their driving to nearby places, reported less difficulty believing they would become a nondriver, and expected to continue driving three years less than non-planners. These findings suggest that drivers’ perceived nearness to driving cessation impacts planning for future transportation needs, and existing perceived benefits of planning may provide leverage to motivate action.

## 1. Introduction

According to the Centers for Disease Control and Prevention (CDC), older adults are expected to make up at least 20% of the United States population by 2050 [1]. In 2014, 79% of Americans 70 and older were licensed drivers, up from 55% in 1983 [2]. However, for many of these individuals, there will be a time when they stop driving permanently, for a variety of reasons. In addition to decrements in physical health (e.g., visual acuity) and cognition that impair one’s ability to safely operate a motor vehicle [3], former drivers also consider licensing problems and costs of owning an automobile [4]. In the United States, more than half a million people annually transition from driving to being a nondriver [5]. Yet, most communities around the country are ill-equipped to handle such a high transition rate. This is especially true in rural and suburban areas, where most older adults live [6].

Older adults outlive their driving lives by several years. On average, men live an additional six years after driving cessation, women a full decade [5]. Unfortunately, alternatives to driving oneself often do not offset the loss of independence, choice, or identity associated with driving. During these years, former drivers may be dependent on loved ones and/or their communities’ transportation resources to meet their mobility needs, while others simply leave their house less often and take fewer trips compared to current drivers [7].

In addition, there are well-established negative health, social, and financial costs borne by older adults who stop driving. Burkhardt [8] describes the monetary, social, psychological, and emotional impact of being a former driver. Driving cessation is also associated with higher risk of nursing home placement [9] and earlier mortality [10].

These increasing burdens may be unnecessarily magnified, however, by a lack of preparation for a time when they will no longer be driving. In fact, qualitative research on driving reduction and cessation suggest that the majority of older adults do not think about or actively prepare for a nondriving future [11,12]. This lack of preparation, not just the outcomes, may be the reason that many former drivers describe their driving retirement as sudden and upsetting life change [11]. 

Despite the reality that hundreds of thousands of older Americans stop driving every year, we do not currently know how older drivers prepare or consider a nondriving future, or how planning affects the individual health and social outcomes associated with driving cessation. We also do not know when people start planning, i.e., when they are middle-aged (50–64) or older adults (65+). Unlike planning for retirement or financial wellbeing in later life, mobility planning is not institutionalized as part of a job or phase of life. As a result, little is known about how people think about or prepare for a future when they are no longer driving.

The following quantitative descriptive research study aimed to fill these knowledge gaps by achieving five aims.

Aim 1: To quantitatively measure planning for future transportation needs among middle-aged and older drivers.
Aim 2: To examine the ways (behaviors) in which drivers seek and utilize information about safe driving and transportation alternatives.
Aim 3: To explore beliefs held by drivers about how useful planning for a nondriving future is and in what ways planning could benefit them.
Aim 4: To measure if drivers intend to do additional planning for their future mobility needs.
Aim 5: To compare individual characteristics and group differences in planning among subgroups (age, self-reported health, gender, race, employment status, and relationship status) to identify correlates and patterns of planning for future transportation needs.

## 2. Materials and Methods

### 2.1. Recruitment

Participants for this study were drawn from two volunteer registries, one through the Claude D. Pepper Older Americans Independence Center (University of Michigan Geriatrics Center, Ann Arbor, MI, USA) and the other the Healthier Black Elders Center (Wayne State University/Michigan Center for Urban African American Aging Research; MCUAAAR, Ann Arbor, MI, USA). Since driving status was not a screening question for either registry, the sole inclusion criterion was age-based (55–84), in order to capture the planning behaviors and expectations of drivers both before, and during, the driving reduction and cessation process. Most drivers are still driving at age 55 with little to no restrictions, while most 84-year-olds have had to actively reduce or adapt their driving in response to physical, cognitive, and social changes. Together, there were 2210 age-eligible volunteers between the two registries. Participants from the Pepper Center registry were largely White, whereas almost all those in the MCUAAAR list were Black. After screening for age (55–84), the registries provided names and contact information for 1322 potential respondents (185 Pepper Center, 1137 MCUAAAR). Since potential participants were being sent questionnaire packets without prior contact, a $2 bill was included as a pre-incentive, with a $20 post-incentive gift card sent to people who returned a completed survey. 

With 874 surveys completed and returned, the overall response rate was 67.8%, after the denominator adjusted for 33 invalid addresses that resulted in survey packets returned to sender without being opened (1322 − 33 = 1289). Of the 1,104 potential MCUAAAR participants, the response rate was 56.5% (*n* = 689). From the Pepper Center registry, *n* = 174 respondents returned surveys, or 94.1%. Two respondents returned their surveys completed, but with the identification number removed, leaving their registry origin unknown.

In order to be eligible for inclusion in the analyses below, respondents had to be currently able to drive, as well as a recent driver (in the past 30 days). Nearly 80% (*n* = 689) of the total sample (*n* = 872) reported being currently able to drive. Of respondents who were able to drive, 624 had also driven recently, resulting in a final analytical sample representing 71.6% of the original respondents. Nearly 70% of MCUAAAR respondents were included (*n* = 480/689), and 82.2% of Pepper Center respondents (*n* = 143/174). One respondent was of unknown registry origin. It is unclear why there was a difference in the response rates between participants in the two registries. 

### 2.2. Instrument

The 18 page, paper and pencil survey covered seven topics, including respondents’ current transportation use; driving history, experiences, and planning; functional health; and demographic characteristics (supplementary). The topics and items were identified from qualitative interviews with older adults (drivers and nondrivers), as well as expert stakeholders in positions or fields relevant to older adults, e.g., geriatrician, police officer, elder law attorney, and adult child of an older driver. The present analysis focuses exclusively on two sections, which measured respondents’ past, current, and future planning for their future transportation needs, as well as current driving context and perceived susceptibility to driving cessation, i.e., level of difficulty believing they may become a nondriver in the future.

### 2.3. Measurement

#### 2.3.1. Planning Variables

The survey instrument contained several questions designed to capture the different dimensions of planning for future mobility needs (Table 1). These included two items to measure self-reported overall planning level for future transportation needs; a global measure, and one specific to a nondriving future. To identify specific behaviors that underlie the concept of planning, respondents also reported how much they had thought about a future when they had reduced or stopped driving completely (thinking); common sources of information (i.e., family and friends, events, or literature) where drivers most frequently learn about the realities and resources of driving in later life (information gathering); and tangible steps respondents take in planning, such as sharing plans with others, writing them down, developing and practicing plans (concrete action).

The survey also measured respondents’ planning beliefs, i.e., the perceived benefits of planning for their future mobility, as well as their intention to do more planning around transportation transitions in the future. Planning beliefs were separated into logistical and emotional. The final variable for the following analyses asked if respondents intended to plan more in the future, regardless of how much they had planned previously.

Response options for most planning items were 5-point Likert scales numbered ascendingly, 0–4, with only the two endpoints anchored by labels (i.e., 0—None/4—All, 0—Not at All/4—A Lot, 0—Not at All/4—Very). The exceptions were Concrete Action items, which ranged from 0 to 3, with each point accompanied by a text anchor (None, A Little, Some, or A Lot). 

Given skewed distribution, the primary outcome, level of nondriving planning, was collapsed into three levels: none (0), low (1–2), and high (3–4).

#### 2.3.2. Driving Context Variables

Given the age range of the sample, it was important to measure specific experiences that may motivate thinking or planning for a nondriving future. Driving fewer days per week, limiting driving geographically, events that made them question their driving skills, and not having a car available when needed, may sensitize middle-aged and older drivers to the reality and challenges of a more imminent nondriving future, thus increasing their planning. Conversely, having difficulty imagining a future where one has ceased driving may be a way to distance oneself from the possibility of driving cessation, which may cause current drivers to avoid the topic of future driving cessation, much less plan for it.

Respondents were asked about their current driving behaviors, specifically driving frequency (average days/week they drove over the past year), if they currently limited their driving to nearby places (yes/no), and whether they had access to a car when they needed one (yes/no). The number of years respondents expect to continue driving was also documented, as well as events in the past year that made respondents consider changing their driving, (e.g., car accident/collision, health issue, or conversation with others about their driving). Fifteen drivers reported unrealistic expectations about how many years they expected to continue driving (80–99 more years of driving), so a maximum value of 50 years of expected driving life remaining was imposed given the age range of participants to adjust for the *n* = 15 drivers who reported unrealistic expectations (80–99 more years of driving). An additional item assessed how much difficulty respondents experienced believing they would become a nondriver someday (Not at All (0)–A Lot (4)). 

#### 2.3.3. Contextual/Demographic Variables

Individual-level demographic information included current age (years); race (self-identification as Black, White, and/or other); gender (male/female); education (less than high school, high school, some college, college graduate, some graduate/professional school, master’s/professional degree, or doctorate); self-reported health (excellent, very good, good, fair, or poor); employment (working, not working, and retired); and relationship status (single/never married, married/domestic partnership, divorced/separated, or widowed, collapsed into partnered (married/domestic partnership) or not (single/never married, divorced/separated, or widowed).

Measured at the household level were two additional variables, annual household income and urbanicity. Income was collected through seven ordinal categories: less than $10,000; $10,000 to $14,999; $15,000 to $24,999; $25,000 to $49,999; $50,000 to $99,999; $100,000 to $149,999; $150,000 to above. Urbanicity, or the density of the areas in which participants live, was self-reported as urban (city), suburban, or rural.

### 2.4. Analytical Approach

We used univariate analyses to determine frequencies, distributions, and types of mobility planning, as well as the direction and strength of the relationship. In addition to reporting the interval-level responses, we also collapsed the planning measures into three levels: none (0), low (1–2), or high (3–4), to compare different levels of planning among subgroups. We used bivariate analyses to test for statistically significant differences in planning (none/low/high) between subgroups, i.e., chi-square tests of difference by gender (male/female), race (White/Black), relationship status (partnered/not partnered), working status (working/not working), health (poor–fair/very good–excellent) and age groups (middle-aged, 53–64, compared to older, 65+ drivers); *t*-tests to compare planning groups by averages of continuous measures (age, driving frequency, number years expected to continue driving, and difficulty believing they could become a nondriver). 

Totals for individual items do not all equal full sample size (*n* = 624) due to missing data. Valid percentages are reported. Missing data were excluded pairwise. Estimates with *p* ≤ 0.05 were considered statistically significant. Analyses were performed using SPSS version 24 (Chicago, IL, USA).

### 2.5. Ethical Considerations

This study was reviewed and granted exempt status approval (HUM00097845) by the University of Michigan Health Sciences and Behavioral Sciences Institutional Review Board (IRB-HSBS) prior to commencing recruitment and data collection. The lead author also answered a series of questions concerning the applicability, appropriateness, and participant protection of this study in order to gain approval of the community advisory board (CAB) before being allowed access to the MCUAAAR/HBEC registry.

## 3. Results

### 3.1. Sample Description

Respondents (*n* = 624) ranged in age from 53 to 92 years old, averaging 72.0 ± 7.1 years (Table 2). Most respondents were older drivers, 65 or older (*n* = 507, 81.3%), with the 16.5% (*n* = 103) younger than 65 categorized as middle-aged drivers. Four respondents were outside the original age range, with two younger than 55 and two above 84 years, possibly due to registry errors in birth dates or individuals other than the intended respondents completing and returning the surveys. 

Overall, the sample was primarily Black (73.9%, *n* = 461) and female (83.1%, *n* = 497). A third of respondents were currently married or in a domestic partnership (36.4%, *n* = 221). On average, the sample was fairly healthy and educated, with nearly half in either Very Good or Excellent health (48.1%, *n* = 296), and college graduates (48.4%, *n* = 416). Over three-quarters were retired (79%, *n* = 490); only 15% (*n* = 93) were employed. Annual household incomes were mostly under $50,000: 40.4% (*n* = 231) made between $25,000 and $49,999, 17.3% (*n* = 99) between $15,000–$24,999, and 11.0% (*n* = 63) less than $15,000. 

The majority of respondents were located in urban environments (66.3%, *n* = 400), compared to a quarter of the sample in suburbs (*n* = 169; Table 2). There was a strong overlap between race and urbanicity as a result of the two volunteer registries from which respondents were recruited, with 78.4% (*n* = 349) of Black participants living in urban environments, compared to just 27.2% (*n* = 36) of White participants. White participants were more likely to live in suburban areas (53.5%, *n* = 71) than Black participants (38.0%, *n* = 169).

### 3.2. Driving Context

Drivers in this sample drove frequently, averaging 5.5 days per week. The vast majority of the sample had a car available when they needed one (98.4%, *n* = 612). Just under a quarter reported limiting their driving to nearby places (22.3%, *n* = 138). Driving participants ranged widely on how difficult they found believing they could someday be nondrivers. Overall, three-quarters of the drivers had difficulty with the concept (74.2%, *n* = 457), averaging 2.1 ± 1.6 on the 0 (Not at All Difficult)–4 (Very Difficult) range. 

In terms of perceived immediacy of a nondriving future, respondents expected to continue driving on average 14.8 ± 8.3 years. One in five (*n* = 94) expected to stop within the next decade. Nearly 15% of drivers (*n* = 68) reported that they expected to drive for 20 years or more. In the past year, 16.1% (*n* = 97) of respondents had experienced at least one health or driving-related event that made them consider changing their driving.

### 3.3. Planning Results

#### 3.3.1. Aim 1: Current Levels of Planning

Overall, drivers in this sample had done very little planning for their future transportation needs (Table 3). Over half of the sample reported they had not planned at all for their general future transportation needs (57.2%, *n* = 345), nor for a possible nondriving future (57.9%, *n* = 361). In addition, only 10.9% (*n* = 66) reported having high levels of planning for their future transportation needs generally. The proportion was even smaller for planning for a nondriving future specifically, with 7.7% (*n* = 48) of respondents endorsing one of the two highest planning levels.

#### 3.3.2. Aim 2: Specific Planning Behaviors

A large group of drivers had not thought about a future where they reduced (30.8%) or stopped driving (41.5%; Table 4). However, nearly a quarter of respondents reported high levels (3–4) of planning for a future with less driving (*n* = 149), compared to only 15% who planned the same amount for a future with no driving (*n* = 96).

In this sample, few drivers sought out information or opportunities to discuss their current driving safety or ways to remain mobile if (or when) they stop driving permanently (Table 4). Slightly more than half of respondents (52.3%, *n* = 322) had read about safe driving for older adults in magazines, brochures, guides, or other sources. Fewer had talked to friends or others to get ideas for their nondriving futures (41.6%, *n* = 218), and only 35.3% (*n* = 218) had attended meetings, lectures, or classes to learn information about aging and driving.

The levels of active planning behaviors corresponded with little planning overall. Most drivers reported doing none of the four concrete actions. Sharing plans was the most common planning action among respondents, with 38% (*n* = 182) having told others about their transportation plans. Only 30% learned about the routes, schedules and other logistical details of getting rides with others or on public transportation. That is twice the number of respondents who wrote their plans down (15.2%, *n* = 93), and a third more than those who practiced their plans (19.1%, *n* = 117). 

#### 3.3.3. Aim 3: Planning Beliefs

Despite having low levels of planning, middle-aged and older drivers believed there were benefits to planning for their transportation future (Table 5). More than 80% of respondents reported that thinking now about a time when they were no longer driving would help them to meet their future transportation needs, as well as transition emotionally to being a nondriver. One in five drivers believed they would benefit “a lot,” the highest level, for both. 

#### 3.3.4. Aim 4: Intention to Plan

Correspondingly, 85% of the sample reported that they intended to do more mobility planning in the future. A third of respondents projected planning at a midpoint between no planning and a lot of planning (33.4%, *n* = 205). About 15% of the sample were on either end of the scale, intending to plan no more (14.8%, *n* = 91), or a lot more (15.3%, *n* = 94).

#### 3.3.5. Aim 5: Subgroup Differences in Planning

##### Demographic Comparisons

Drivers who had planned for a nondriving future were slightly, albeit significantly, older on average than non-planners (*t*(608) = −2.4, *p* = 0.02; Table 6). Non-planners were statistically significantly more likely to be employed full- or part-time than planners (*X*^2^ (1, *n* = 624) = 5.39, *p* = 0.02). Drivers who reported being in poor or fair health were marginally more likely to have planned for a nondriving future when compared to those in very good or excellent health (*X*^2^ (1, *n* = 379) = 3.38, *p* = 0.07). There were no statistically significant differences in whether or not drivers had planned for a nondriving future (yes/no) based on age group (middle-aged or older drivers; *X*^2^ (1, *n* = 610) = 1.4, *p* = 0.24); gender (*X*^2^ (1, *n* = 595) = 1.1, *p* = 0.30), race (Black/White; *X*^2^ (1, *n* = 594) = 2.0, *p* = 0.16); or relationship status (partnered/not partnered; *X*^2^ (1, *n* = 607) = 3.1, *p* = 0.08).

The majority of respondents who had planned for a nondriving future were categorized as low-level planners (81.7%; Table 6). Among planners, a higher proportion of Black drivers reported high levels of planning (3–4) compared to White drivers (*X*^2^ (1, *n* = 610) = 4.6, *p* = 0.03). There was not a significant difference in mean age between high and low planners (*t*(255) = 0.99, *p* = 0.32), nor between age groups (*X*^2^ (1, *n* = 257) = 0.00, *p* = 0.98); gender (*X*^2^ (1, *n* = 253) = 1.3, *p* = 0.25); relationship status (*X*^2^ (1, *n* = 259) = 2.44, *p* = 0.12); employment status (*X*^2^ (1, *n* = 263) = 0.02, *p* = 0.88); or dichotomized health status (*X*^2^ (1, *n* = 154) = 0.76, *p* = 0.39). 

##### Driving Context Comparisons

Planners averaged significantly fewer driving days per week in the past year compared to drivers who had not planned at all (*t*(609) = 2.2, *p* = 0.03), and were significantly less likely to drive daily (*X*^2^ (1, *n* = 603) = 5.2, *p* = 0.02; Table 7). Similarly, respondents were more likely to plan if they limited their driving to nearby places (52.2% vs. 39.3%; *X*^2^ (1, *n* = 619) = 7.3, *p* = 0.01). No differences in planning were found based on car availability (*X*^2^ (1, *n* = 622) = 0.02, *p* = 0.89). 

Compared to planners, respondents who had not planned at all for a nondriving future had significantly more difficulty believing they would become a nondriver in the future, averaging 2.3 ± 1.6 vs. 2.0 ± 1.5 on the Not at All Difficult (0) to Very Difficult (4) scale (*t*(614) = 2.4, *p* = 0.02; Table 7). This is likely related to perceived nearness of driving cessation: planners expected to stop driving in the next 13.3 ± 8.0 years on average, compared to 15.9 ± 8.4 years for non-planners (*t*(469) = 3.5, *p* > 0.01). In the past year, over half (55.1%) of planners had experienced an event that made them consider changing their driving, significantly more than the 39.9% reported by non-planners (*X*^2^ (1, *n* = 601) = 4.6, *p* = 0.03). 

Planners who limit their driving to nearby places are significantly more likely to report high levels of planning compared to those who do not limit (*X*^2^ (1, *n* = 261) = 4.2, *p* = 0.04; Table 7). Levels of planning (high/low) did not differ among planners by number of driving days (*t*(256) = 0.1, *p* = 0.90), if they drive daily (*X*^2^ (1, *n* = 253) = 0.00, *p* = 1.0), or by car availability (*X*^2^ (1, *n* = 262) = 0.1, *p* = 0.73), although there were few respondents (*n* = 4) who did not have a car available between both groups. In addition, high- and low-level planners did not differ in how much difficulty they had believing they would be a nondriver one day (*t*(258) = 0.9, *p* = 0.35), how many years they expected to continue driving (*t*(197) = 0.8, *p* = 0.46), or experiencing events that made them consider changing their driving (*X*^2^ (1, *n* = 251) = 1.3, *p* = 0.25).

##### Planning Beliefs Comparisons

In this sample, drivers who planned endorsed stronger beliefs about how planning now would benefit them in a nondriving future. On a scale of 0 (Not at All) to 4 (A Lot), planners averaged significantly greater belief that planning would help them meet their post-cessation transportation needs compared to non-planners (2.4 ± 1.1 vs. 2.0 ± 1.5; *t*(611) = −3.5, *p* > 0.01). Similarly, planners also reported that their actions have a greater impact on easing the emotional transition to nondriver relative to non-planners (2.3 ± 1.1 vs. 2.0 ± 1.5; *t*(609) = −3.2, *p* > 0.01). Among planners, greater beliefs in the practical benefits of preparation were associated with high levels for a nondriving future (2.8 ± 1.1 vs. 2.3 ± 1.1; *t*(256) = −2.8, *p* < 0.01). However, there were no differences in beliefs about emotional benefits of planning between high- and low-level planners (*t*(258) = −1.1, *p* = 0.29). 

## 4. Discussion

Overall, we found that planning for driving cessation among a sample of drivers aged 53–92 years was low across multiple domains. Drivers were unlikely to gather information about older driver safety, to explore the community resources available to them, or to think about a nondriving future. Additionally, it was notable that nearly a third of the sample found it difficult to believe they might become a nondriver. Most reported that they had not even considered the possibility of a future where they drive less, or not at all.

However, drivers’ perceived nearness to driving cessation appears to impact planning levels. In this sample, planning for a nondriving future was associated with several characteristics previously shown to predict driving cessation, including increased age, reduced driving frequency, limiting driving to nearby places, and health or driving-related events that drew attention to their driving abilities. Planners had less difficulty believing they could become a nondriver someday, and in fact, expected to stop nearly three years sooner than drivers who had not planned at all. Interestingly, demographic characteristics (i.e., gender and race) did not differ between planners and non-planners. Taken together, these findings suggest that middle-aged and older drivers do not start to prepare for a nondriving future until that future feels relatively imminent for them personally. 

If planning for nondriving futures is the most beneficial for those at the highest risk, our sample overrepresents those who would benefit the most from preparation. Although male drivers are more resistant to driving cessation, women did not plan more for a nondriving future in the present study. Similarly, previous research has shown that people of color stop driving earlier than men and White drivers [13,14], however, Black drivers were not more likely to have planned either. Furthermore, the majority of respondents lived in and around Detroit, an especially challenging city for nondrivers [15]. Unfortunately, even drivers in this sample appear somewhat lukewarm to the concept and nearly inactive in practice. These findings suggest that the characteristics predictive of earlier driving cessation are not necessarily associated with whether or not a driver plans for a nondriving future.

There are several possible reasons that older adults may avoid the topic of driving cessation. For example, research demonstrates that for some older adults, especially those with few transportation mobility limitations, the topic represents “an unacceptable thought related to some distant future” [11] (p. 42). Other research indicates that older adults in most parts of the United States perceive a lack of alternatives to driving oneself, and generally assume that they will rely on friends/family to take them places after cessation [7].

However, over 80% of respondents in this study believed that preparing for a nondriving future would improve their futures, both in terms of meeting their transportation needs and transitioning emotionally if they stopped driving. Similarly, 85% reported that they intended to do more mobility planning in the future. Previous research found that older drivers’ general awareness of mobility limitations does not automatically translate into personal contemplation or action [16]. Leveraging planning beliefs that preparation can benefit them in the future may be one way to bridge this gap.

Future research is needed to comprehensively explore the concept and predictors of planning for future transportation needs among middle-aged and current. Other directions include determining how preparation for a nondriving future effects the process of driving reduction and cessation. Another crucial link is if planning translates into improved health, social, and community mobility outcomes among former drivers. 

Additionally, identifying barriers to planning is crucial, as there are many logical but distinct reasons why current drivers might not think about or plan for a nondriving future. Although drivers may see benefits to planning for a nondriving future, they may not believe that they will ever be in that position. As such, planning is unnecessary, at best, and wasteful of precious time and energy, at worst. A second potential explanation is that completing the survey acted as an intervention of sorts, cueing people to action on a topic they had not considered or cared about prior to participating. In this scenario, raising consciousness about preparing for nondriving future may be a key to motivating people to plan by the simple implication that one has the ability to prepare for such a time. A third possibility is that drivers avoid thinking or talking about any issue that invokes the specter of driving cessation, even if there may be benefits to doing so. It is crucial to identify the barriers to planning, in order to effectively promote preparation. Further qualitative and quantitative data collection is needed to address these remaining gaps.

As with any research, the present study has its limitations. First, due to the recruitment approach, the respondents were not nationally representative; as such, the findings described herein are not necessarily generalizable to all middle-aged and older drivers in the United States. A second limitation is the cross-sectional nature of the data, which limits our assessment of change in beliefs and behaviors over time. Because of this, these data cannot tell us how (or if) beliefs and behaviors around transportation planning change among drivers over 50 as they get older, or if intention to plan leads to more planning. 

Finally, it is reasonable to question the measurement, given the novel items and very low averages of planning behaviors captured. In other words, survey items may not have asked about the most relevant aspects of planning, or accurately described the ways middle-aged and older drivers are thinking about or preparing for a time when they are no longer driving. However, the older driver literature is laden with reasons the topic and reality of driving cessation are uncomfortable, at best, and taboo at worst.

However, the strengths of the data far outweigh these limitations. There have been few previous studies that directly assess how much drivers prepare for mobility transitions; the topic is glanced upon in some qualitative work on older drivers and driving cessation. This unique, novel dataset not only explores several facets of mobility planning among middle-aged and older drivers, it does so with a large sample. These individuals were not only numerous, but primarily identified as Black, a valuable voice commonly missing outside of huge federal surveys.

Despite the popular beliefs of a driving future replete with autonomous vehicles that might solve transportation challenges faced by older adults, the reality is that issues related to driving cessation (and the transportation disability it causes) are not going to be solved anytime soon. For at least the next several decades, there will be a critical need and immense value in improving the process and outcomes of driving retirement. Our results provide crucial insights regarding both the paucity of planning behaviors currently being undertaken by middle-aged and older drivers, and the strength of their beliefs that planning might be beneficial. Understanding both the barriers and facilitating factors in planning can inform interventions that build on current drivers’ beliefs about how planning can benefit them, thereby setting them up for improved outcomes when driving cessation does, in fact, occur.

## Figures and Tables

**Table 1:** Planning-Related Measures.

Category	Survey Items
Current Level of Planning	How much have you planned for your possible future transportation needs? This includes how you may need to change or adapt how you get around outside your home and new needs for transportation you may have in the future.
	How much have you planned for a time in the future when you may no longer be driving?
Concrete Planning Actions	How much have you thought about a possible future time when you are still driving, but drive less than you currently do?
	How much have you thought about a future time when you are no longer driving at all?
	How much or often have you talked to friends or others to get ideas or information for your possible future transportation needs?
	How many meetings, lectures, or classes have you attended to learn information about aging and driving?
	How much information about safe driving for older adults have you sought out from magazine articles, brochures, guides, or other sources (either printed or on the internet)?
	How much have you done each of the following actions to make your future transportation plans more concrete?
	Figure out the routes, schedules, and other logistical details of getting rides with others or on public transit.
	Write your plan down.
	Practice the plan to become more comfortable or familiar with it.
	Tell other people about your plan.
Planning Beliefs	How much would thinking now about a time when you are no longer driving help you meet future transportation needs?
	How much would thinking now about a time when you are no longer driving help make a future transition to nondriver easier emotionally?
Planning Intention	Regardless of how much transportation planning you have or haven’t done, how much planning about your transportation do you intend to do in the future?

**Table 2:** Characteristics of Sample.

Demographic Characteristic	All	Age Group		Race		Gender	
	*n* (%)	Middle	Older	Black	White	Female	Male
Age 72.0 (7.1) years	610	61.2 (2.8)	74.2 (5.5)	71.8 (6.8)	72.9 (8.1)	71.8 (6.7)	72.6 (7.8%)
Middle-Aged (53–64)	103 (16.5%)	--	--	72 (15.7%)	29 (21.6%)	84 (17.0%)	18 (18.4%)
Older Drivers (65–92)	507 (83.1%)	--	--	387 (84.3%)	105 (78.4%)	410 (83.0%)	80 (81.6%)
Race							
Black	461 (73.9%)	72 (69.9%)	387 (76.6%)	--	--	394 (79.9%)	50 (51.0%)
White	135 (21.6%)	29 (28.2%)	105 (20.7%)	--	--	89 (18.1%)	45 (45.9%)
Other	25 (4.0%)	2 (1.9%)	13 (2.6%)	--	--	10 (2.0%)	3 (3.1%)
Gender (Female)	497 (83.1%)	84 (82.4%)	410 (83.7%)	394 (88.7%)	89 (66.4%)	--	--
Education							
High School Diploma or Less	96 (15.7%)	13 (12.7%)	83 (16.4%)	85 (18.5%)	10 (7.5%)	72 (14.5%)	17 (17.3%)
Some College	200 (32.7%)	32 (31.4%)	166 (32.7%)	155 (33.8%)	36 (26.9%)	171 (34.5%)	24 (24.5%)
College Degree	108 (17.7%)	40 (39.2%)	127 (25.1%)	81 (17.6%)	27 (20.1%)	134 (27.1%)	28 (28.6%)
Master’s/Professional Degree	133 (21.8%)	16 (15.7%)	117 (23.1%)	91 (19.8%)	39 (29.1%)	108 (21.8%)	25 (25.5%)
Doctorate	15 (2.5%)	1 (1.0%)	14 (2.8%)	10 (2.2%)	4 (3.0%)	10 (2.0%)	4 (4.1%)
Employment Status							
Working	93 (14.9%)	45 (43.7%)	48 (9.5%)	61 (13.3%)	28 (20.9%)	75 (15.1%)	17 (17.3%)
Retired	490 (78.5%)	47 (45.6%)	433 (85.4%)	368 (80.0%)	102 (76.1%)	397 (79.9%)	73 (74.5%)
Relationship Status							
Single (Never Married)	51 (8.4%)	19 (19.0%)	31 (6.1%)	45 (9.9%)	4 (3.0%)	44 (9.0%)	5 (5.3%)
Married/Domestic Partnership	221 (36.4%)	46 (46.0%)	172 (33.9%)	126 (27.8%	90 (68.2%)	147 (30.1%)	69 (73.4%)
Divorced/Separated	182 (30.0%)	23 (23.0%)	157 (31.7%)	162 (35.8%)	14 (10.6%)	160 (32.7%)	13 (13.8%)
Widowed	153 (25.5%)	12 (12.0)	136 (27.4%)	120 (26.5%)	24 (18.2%)	138 (28.2%)	7 (7.4%)
Self-Reported Health							
Excellent	53 (8.6%)	16 (15.5%)	37 (7.4%)	26 (5.8%)	25 (18.7%)	41 (8.4%)	10 (10.2%)
Very Good	243 (39.5%)	32 (31.1%)	204 (40.8%)	168 (37.2%)	62 (46.3%)	195 (39.8%)	39 (39.8%)
Good	236 (38.4%)	39 (38.2%)	195 (39.0%)	191 (42.3%)	37 (27.6%)	189 (38.6%)	37 (37.8%
Fair	77 (12.5%)	12 (11.8%)	61 (12.2%)	562 (13.5%)	9 (6.7%)	62 (12.5%)	9 (9.2%)
Poor	6 (1.0%)	3 (2.9%)	3 (0.6%)	5 (1.1%)	1 (0.7%)	3 (0.6%)	3 (3.1%)
Annual Household Income							
<$10,000	18 (3.1%)	4 (4.2%)	14 (3.0%)	16 (3.8%)	1 (0.8%)	10 (2.2%)	4 (4.3%)
$10,000–$14,999	45 (7.9%)	9 (9.4%)	35 (7.5%)	43 (10.2%)	2 (1.6%)	39 (8.5%)	3 (3.2%)
$15,000–$24,999	99 (17.3%)	11 (11.5%)	83 (17.8%)	81 (19.3%)	8 (6.2%)	83 (18.1%)	10 (10.8%)
$25,000–$49,999	231 (40.4%)	35 (36.5%)	194 (41.5%)	178 (42.4%)	45 (34.9%)	192 (41.9%)	32 (34.4%)
$50,000–$99,999	132 (23.1%)	26 (25.2%)	105 (22.5%)	76 (18.1%)	53 (41.1%)	96 (21.0%)	35 (37.6%)
$100,000–$149,999	36 (6.3%)	7 (7.3%)	29 (6.2%)	21 (5.0%)	14 (10.9%)	31 (6.8%)	5 (5.4%)
>$150,000	11 (1.9%)	4 (4.1%)	7 (1.5%)	5 (1.2%)	6 (4.7%)	7 (1.5%)	4 (4.3%)
Urbanicity							
Urban (City)	400 (66.3%)	57 (55.9%)	334 (68.2%)	349 (78.1%)	36 (27.5%)	331 (68.5%)	50 (52.1%)
Suburban	169 (28.0%)	33 (32.4%)	134 (27.3%)	90 (20.1%)	70 (53.4%)	128 (26.5%)	36 (37.5%)
Rural	31 (5.1%)	11 (10.8%)	20 (4.1%)	5 (1.1%)	25 (19.1%)	21 (4.3%)	10 (10.4%)

**Table 3:** Drivers’ Levels of Planning for Future Transportation Needs.

Planning Item	All	Age Group		Race		Gender	
	*n* (%)	Middle	Older	Black	White	Female	Male
Planned for future transportation needs							
None (0)	345 (57.2%)	54 (54.0%)	281 (57.5%)	250 (56.4%)	75 (57.7%)	275 (57.4%)	52 (54.2%)
Low (1–2)	192 (31.8%)	39 (39.0%)	150 (29.6%)	143 (32.3%)	43 (33.1%)	151 (31.5%)	36 (37.5%)
High (3–4)	66 (10.9%)	7 (6.8%)	58 (11.9%)	50 (11.3%)	12 (9.2%)	53 (11.1%)	8 (8.3%)
% Any	258 (42.8%)	46 (46.0%)	208 (41.5%)	193 (43.6%)	55 (42.3%)	204 (42.6%)	44 (45.8%)
Planning for nondriving future							
None (0)	361 (57.9%)	65 (63.1%)	288 (56.8%)	257 (55.9%)	84 (62.7%)	281 (56.5%)	61 (62.2%)
Low (1–2)	215 (34.5%)	31 (30.1%)	179 (35.3%)	160 (34.8%)	46 (34.3%)	176 (35.4%)	33 (33.7%)
High (3–4)	48 (7.7%)	7 (6.8%)	40 (7.9%)	43 (9.3%)	4 (3.0%)	40 (8.0%)	4 (4.1%)
% Any	263 (42.1%)	38 (36.9%)	219 (43.2%)	203 (44.1%)	50 (37.3%)	216 (43.3%)	37 (37.8%)

**Table 4:** Drivers’ Planning Behaviors for Future Transportation Needs.

Planning Behavior	All	Age Group		Race		Gender	
	*n* (%)	Middle-Aged	Older	Black	White	Female	Male
Thought about future with reduced driving							
0—Not at All	192 (30.8%)	29 (28.2%)	157 (31.0%)	134 (29.1%)	46 (34.3%)	146 (29.4%)	32 (32.7%)
1	120 (19.2%)	24 (23.3%)	94 (18.5%)	76 (16.5%)	37 (27.6%)	96 (19.3%)	21 (21.4%)
2	163 (26.1%)	31 (30.1%)	129 (25.4%)	130 (28.3%)	26 (19.4%)	134 (27.0%)	24 (24.5%)
3	108 (17.3%)	14 (13.6%)	92 (18.1%)	86 (18.7%)	20 (14.9%)	90 (18.1%)	16 (16.3%)
4—A Lot	41 (6.6%)	5 (4.9%)	35 (6.9%)	34 (7.4%)	5 (3.7%)	31 (6.2%)	5 (5.1%)
% Any	432 (69.2%)	74 (71.8%)	350 (69.0%)	326 (70.9%)	88 (65.7%)	351 (70.6%)	66 (67.3%)
Thought about nondriving future							
0—Not at All	257 (41.5%)	49 (47.6%)	202 (40.2%)	183 (40.0%)	60 (45.5%)	200 (40.5%)	43 (44.8%)
1	159 (25.7%)	40 (38.8%)	113 (22.5%)	106 (23.2%)	42 (31.8%)	131 (26.5%)	23 (24.0%)
2	107 (17.3%)	9 (8.7%)	98 (19.5%)	88 (19.3%)	17 (12.9%)	87 (17.6%)	18 (18.8%)
3	50 (8.1%)	0 (0.0%)	49 (9.8%)	44 (9.6%)	5 (3.8%)	43 (8.7%)	5 (5.2%)
4—A Lot	46 (7.4%)	5 (4.9%)	40 (8.0%)	36 (7.9%)	2 (1.5%)	33 (6.7%)	7 (7.3%)
% Any	362 (58.5%)	54 (52.4%)	300 (59.8%)	274 (60.0%)	72 (54.5%)	294 (59.5%)	53 (55.2%)
Talked to others to get ideas							
0—Not at All	360 (58.4%)	58 (57.4%)	292 (58.3%)	259 (57.2%)	81 (60.9%)	284 (57.8%)	61 (62.9%)
1	114 (18.5%)	24 (23.8%)	89 (17.8%)	80 (17.7%)	30 (22.6%)	92 (18.7%)	19 (19.6%)
2	80 (13.0%)	12 (11.9%)	67 (13.4%)	61 (13.5%)	16 (12.0%)	65 (13.2%)	12 (12.4%)
3	29 (4.7%)	3 (3.0%)	25 (5.0%)	44 (9.6%)	4 (3.0%)	25 (5.1%)	2 (2.1%)
4—A Lot	33 (5.4%)	4 (4.0%)	28 (5.6%)	36 (7.9%)	2 (1.5%)	25 (5.1%)	3 (3.1%)
% Any	256 (41.6%)	43 (42.6%)	209 (41.7%)	194 (42.8%)	52 (39.1%)	207 (42.2%)	36 (37.1%)
Attended meetings, lectures, or classes							
0—Not at All	399 (64.7%)	77 (76.2%)	311 (62.0%)	262 (57.7%)	113 (85.0%)	306 (62.3%)	76 (77.6%)
1	79 (12.8%)	4 (4.0%)	74 (14.7%)	66 (14.5%)	11 (8.3%)	69 (14.1%)	7 (7.1%)
2	71 (11.5%)	11 (10.9%)	58 (11.6%)	64 (14.1%)	5 (3.8%)	56 (11.4%)	12 (12.2%)
3	35 (5.7%)	4 (4.0%)	31 (6.2%)	31 (6.8%)	3 (2.3%)	33 (6.7%)	2 (2.0%)
4—A Lot	33 (5.3%)	5 (5.0%)	28 (5.6%)	31 (6.8%)	1 (0.8%)	27 (5.5%)	1 (1.0%)
% Any	218 (35.3%)	24 (23.8%)	191 (38.0%)	192 (42.3%)	20 (15.0%)	185 (37.7%)	22 (22.4%)
Read magazine articles, brochures, guides or other sources							
0—Not at All	294 (47.7%)	65 (64.4%)	219 (43.7%)	207 (45.7%)	69 (51.9%)	219 (44.8%)	59 (60.2%)
1	139 (22.6%)	20 (19.8%)	117 (23.4%)	87 (19.2%)	47 (35.3%)	109 (22.3%)	26 (26.5%)
2	102 (16.6%)	13 (12.9%)	87 (17.4%)	86 (19.0%)	12 (9.0%)	92 (18.8%)	9 (9.2%)
3	37 (6.0%)	1 (1.0%)	36 (7.2%)	31 (6.8%)	4 (3.0%)	33 (6.7%)	2 (2.0%)
4—A Lot	44 (7.1%)	2 (2.0%)	42 (8.4%)	42 (9.3%)	1 (0.8%)	36 (7.4%)	2 (2.0%)
% Any	322 (52.9)	36 (35.6%)	282 (56.3%)	246 (54.3%)	64 (48.1%)	270 (55.2%)	39 (39.8%)

**Table 5:** Drivers’ Beliefs about Effects of Planning for Future Transportation Needs.

Planning Belief	All	Age Group		Race		Gender	
	*n* (%)	Middle-Aged	Older	Black	White	Female	Male
Help meet transportation needs							
0—Not at All	97 (15.8%)	17 (16.7%)	77 (15.5%)	57 (12.7%)	31 (23.1%)	65 (13.3%)	23 (23.7%)
1	84 (13.7%)	18 (17.6%)	63 (12.7%)	53 (11.8%)	28 (20.9%)	63 (12.9%)	18 (18.6%)
2	184 (30.0%)	31 (30.4%)	149 (30.0%)	137 (30.5%)	36 (26.9%)	151 (30.9%)	30 (30.9%)
3	116 (18.9%)	18 (17.6%)	97 (19.5%)	89 (19.8%)	25 (18.7%)	100 (20.5%)	11 (11.3%)
4—A Lot	132 (21.5%)	18 (17.6%)	111 (22.3%)	113 (25.2%)	14 (10.4%)	109 (22.3%)	15 (15.5%)
% Any	516 (84.2%)	85 (83.3%)	420 (84.5%)	392 (87.3%)	103 (76.9%)	423 (86.7%)	74 (76.3%)
Help emotional transition to nondriver							
0—Not at All	105 (17.2%)	19 (18.6%)	83 (16.7%)	65 (14.5%)	32 (23.9%)	76 (15.6%)	21 (21.4%)
1	82 (13.4%)	12 (11.8%)	68 (13.7%)	52 (11.6%)	24 (17.9%)	64 (13.2%)	15 (15.3%)
2	182 (29.8%)	34 (33.3%)	144 (29.0%)	140 (31.2%)	33 (24.6%)	143 (29.4%)	33 (33.7%)
3	122 (20.0%)	21 (20.6%)	100 (20.1%)	91 (20.3%)	30 (22.4%)	101 (20.8%)	16 (16.3%)
4—A Lot	120 (19.6%)	16 (15.7%)	102 (20.5%)	101 (22.5%)	15 (11.2%)	102 (21.0%)	13 (13.3%)
% Any	506 (82.8%)	83 (81.4%)	414 (83.3%)	384 (85.5%)	102 (76.1%)	410 (84.4%)	77 (78.6%)

**Table 6:** Demographic Comparisons of Planning for Nondriving Future.

Demographic Characteristic	Planned At All		Planning Level	
	Yes	No	Low (1–2)	High (3–4)
Age *M* (*SD*)	72.8 (7.0) *	71.4 (7.1) *	73.0 (7.0)	72.0 (7.3)
Middle-Aged (53–64)	38 (36.9%)	65 (63.1%)	31 (81.6%)	7 (18.4%)
Older (65+)	219 (43.2%)	288 (56.8%)	179 (81.7%)	40 (18.3%)
Gender				
Female	216 (43.5%)	281 (56.5%)	176 (81.5%)	40 (18.5%)
Male	37 (37.8%)	61 (62.2%)	33 (89.2%)	4 (10.8%)
Race				
Black	203 (44.1%)	257 (55.9%)	160 (78.8%) *	43 (21.2%) *
White	50 (37.3%)	84 (62.7%)	46 (92.0%) *	4 (8.0%) *
Relationship Status				
Partnered	84 (38.0%)	137 (62.0%)	73 (86.9%)	11 (13.1%)
Not Partnered	175 (45.3%)	211 (54.7%)	138 (78.9%)	37 (21.1%)
Employment Status				
Working	29 (31.2%) *	64 (68.8%) *	24 (82.8%)	5 (17.2%)
Not Working	234 (44.1%) *	297 (55.9%) *	191 (81.6%)	43 (18.4%)
Health				
Poor/Fair	41 (49.4%)	42 (50.6%)	32 (78.0%)	9 (22.0%)
Very Good/Excellent	113 (38.2%)	183 (61.8%)	95 (84.1%)	18 (15.9%)

* *p* ≤ 0.05.

**Table 7:** Demographic Comparisons of Planning for Nondriving Future.

Driving Context	Planned At All		Planning Level	
	Yes	No	Low	High
Driving Frequency (Days Per Week) *M* (*SD*)	5.3 (1.8) *	5.6 (1.7) *	5.3 (1.8)	5.2 (1.7)
Daily Driver	88 (36.4%) *	154 (63.6%) *	72 (81.8%)	16 (18.2%)
Non-Daily Driving	165 (45.7%) *	196 (54.3%) *	135 (81.8%)	30 (18.2%)
Limits Driving to Nearby Places				
Yes	72 (52.2%) **	66 (47.8%) **	53 (73.6%) *	19 (26.4%) *
No	189 (39.3%) **	292 (60.7%) **	160 (84.7%) *	29 (15.3%) *
Car Available When Needed				
Yes	258 (42.2%)	354 (57.8%)	211 (81.8%)	47 (18.2%)
No	4 (40.0%)	6 (60.0%)	3 (75.0%)	1 (25.0%)
Difficulty Believing Will Become Nondriver *(0—Not at All–4—A Lot) M (SD)*	2.0 (1.5) *	2.3 (1.6) *	2.0 (1.5)	1.8 (1.6)
Expected Years of Driving Remaining *M* (*SD*)	13.3 (8.0) **	15.9 (8.4) **	13.4 (7.9)	12.3 (9.4)
Experienced Event(s) in Past Year That Made Them Consider Changing Driving				
Yes	50 (51.5%) *	47 (48.5%) *	38 (76.0%)	12 (24.0%)
No	201 (39.9%) *	303 (60.1%)*	167 (83.1%)	34 (16.9%)

* *p* ≤ 0.05; ** *p* ≤ 0.01.

## Supplementary Materials

The following are available online at http://www.mdpi.com/2308-3417/3/2/19/s1, Table S1: Survey about Transportation.
