# Supplementary material for: Planning for a Nondriving Future: Behaviors and Beliefs among Middle-Aged and Older Drivers
Source: Geriatrics (Basel). 2018 Apr 13;3(2):19. doi: 10.3390/geriatrics3020019 (PMC5914519; doi:10.3390/geriatrics3020019)
Supplement: Supplementary file 1 [file geriatrics-03-00019-s001.pdf]

Survey about Transportation

DIRECTIONS: Please respond to as many questions as possible. If you are unsure, please pick the closest response or guess.

To begin, we'd like to ask you a few questions about how you get wherever you need to go, both now and what you expect in the future.

1. *How satisfied are you with your current transportation mobility? In other words, how easily can you get where you need or want to go?*

   Not at All Satisfied ☐ ☐ ☐ ☐ ☐ **Very Satisfied**

2. *How much are your current transportation needs being met using each of the following transportation methods?*

a. *DRIVING YOURSELF*	☐ ☐ ☐ ☐ ☐ **None** **All**
b. *RIDES WITH OTHER DRIVERS* (family, friends, etc.)	☐ ☐ ☐ ☐ ☐ **None** **All**
c. *BUSES*	☐ ☐ ☐ ☐ ☐ **None** **All**
d. *TAXIS/CABS*	☐ ☐ ☐ ☐ ☐ **None** **All**
e. *MASS TRANSPORT* (light rail, trains, etc.)	☐ ☐ ☐ ☐ ☐ **None** **All**
f. *SPECIALIZED TRANSPORT* (medical transport, disabled/senior shuttles, etc.)	☐ ☐ ☐ ☐ ☐ **None** **All**
g. *WALKING* (for transportation, NOT for enjoyment or exercise exclusively)	☐ ☐ ☐ ☐ ☐ **None** **All**
h. *"E-HAIL" APPS* (such as Uber or Lyft) on a smartphone or tablet	☐ ☐ ☐ ☐ ☐ **None** **All**
i. OTHER Please specify:________________________	☐ ☐ ☐ ☐ ☐ **None** **All**

3. *How much do you know about "E-Hail" apps (such as Uber or Lyft) for smartphones or tablets that can help arrange rides?*

☐ None ☐ A Little ☐ Some ☐ A Lot

4. *How much have you used "E-Hail" apps (such as Uber or Lyft) on a smartphone or tablet to arrange rides?*

☐ None ☐ A Little ☐ Some ☐ A Lot

5. *How much have you planned for your possible future transportation needs? This includes how you may need to change or adapt how you get around outside your home and new needs for transportation that you may have in the future.*

Not at All ☐ ☐ ☐ ☐ ☐ **A Lot**

How much or often have you talked to friends or others...

**6.** *...to get ideas or information for your possible future transportation needs?*	☐ ☐ ☐ ☐ ☐ **Not at All** **A Lot**
**7.** *...about how <u>they</u> get around without driving?*	☐ ☐ ☐ ☐ ☐ **Not at All** **A Lot**

8. How much have you done each of the following actions to make your future transportation plans more concrete?

a. *Tell other people about your plans*	☐ **None**	☐ **A Little**	☐ **Some**	☐ **A Lot**
b. *Write your plans down*	☐ **None**	☐ **A Little**	☐ **Some**	☐ **A Lot**
c. *Figure out the routes, schedules, and other logistical details of getting rides with others or on public transit*	☐ **None**	☐ **A Little**	☐ **Some**	☐ **A Lot**
d. *Practice the plan to become more comfortable or familiar with it*	☐ **None**	☐ **A Little**	☐ **Some**	☐ **A Lot**

9. *Have you <u>ever</u> been a driver?* ☐ Yes ☐ No

↓

*If you have **<u>NEVER</u>** been a driver, please skip to page 12, question 48.*

Next, we have more questions about your driving experiences.

10. *How old were you when you learned to drive?* _____ years old

11. How experienced do you feel you are as a driver?

    Not at All Experienced ☐ ☐ ☐ ☐ ☐ **Very Experienced**

12. *For how many years did you drive* <u>*intensely*</u> *on a regular basis, that is, driving frequently and/or long distances for your work or personal life?* _____ years

13. *At any point in your driving history, have you modified your driving in any of the following ways* (please select all that apply)*:*

    ☐ Drive only with others in the car
    ☐ Avoid left-hand turns
    ☐ Avoid peak traffic hours
    ☐ Stay within familiar areas
    ☐ Temporarily been unable to drive
    ☐ Drive slower than you used to
    ☐ Drive only during daylight
    ☐ Avoid busy intersections
    ☐ Avoid highways/interstates

    ☐ Other (please describe):

For the next set of questions, we will focus on people or places where you might get information about safe driving.

**14.** *How many meetings, lectures, or classes have you attended to learn information about aging and driving?*	☐ ☐ ☐ ☐ ☐ **None** **A Lot**
**15.** *How much information about safe driving for older adults have you sought out from magazine articles, brochures, guides, or other sources (either printed or on the Internet)?*	☐ ☐ ☐ ☐ ☐ **None** **A Lot**
**16.** *Regardless of how much transportation planning you have or haven't done, how much planning about your transportation do you intend to do in the future?*	☐ ☐ ☐ ☐ ☐ **None** **A Lot**

17. *Have you talked to family, friends, or others about how they plan to get around if they stop driving?*

Not at All ☐ ☐ ☐ ☐ ☐ **A Lot**

18. *Are you responsible for anyone else's transportation?*

☐ Yes ☐ No

↓

IF YES, please describe:

19. *How many drivers live with you (not including yourself, if you currently drive)?*_____ drivers

20. *Are you currently able to drive?*

☐ Yes ☐ No

↓

*IF NO, how many years has it been since the last time you drove?*_____ years

↓

IF NO, why did you stop driving?

If you are ***NOT CURRENTLY*** *able to drive, please skip to page 12, question 48.*

21. *Do you have a car available to use when you need one?* ☐ Yes ☐ No

22. *Do you limit your driving to nearby places?* ☐ Yes ☐ No

23. *Do you drive on longer trips?* ☐ Yes ☐ No

24. *In the past year, how many days (on average) did you drive each week?*

_____ days/week

25. *How difficult is it for you to believe that you may become a nondriver someday?*

Not at All Difficult ☐ ☐ ☐ ☐ ☐ **Very Difficult**

**26.** *How stressful is driving for you currently?*	☐ ☐ ☐ ☐ ☐ **Not at All** **Very**
**27.** *Whether or not driving is stressful to you, how enjoyable is it for you currently?*	☐ ☐ ☐ ☐ ☐ **Not at All** **Very**
**28.** *If you were no longer able to drive, how satisfied do you think you would be with your transportation mobility?*	☐ ☐ ☐ ☐ ☐ **Not at All** **Very**

How much would thinking now about a time when you're no longer driving…

**29.** *...help you to meet future transportation needs?*	☐ ☐ ☐ ☐ ☐ **Not at All** **A Lot**
**30.** *...help make a future transition to nondriver easier emotionally?*	☐ ☐ ☐ ☐ ☐ **Not at All** **A Lot**

31. *When do you think you will stop driving completely?*

32. *In the past year, have you experienced any events <u>that made you consider changing your driving</u>?*

☐ Yes ☐ No

↓

<u>IF YES</u>, please mark what kind of events occurred (please select all that apply)*:*

☐ Car accident or collision

☐ Someone you know stopped driving

☐ A conversation about your driving

☐ Backing up into objects

☐ Finding unexplained dents or dings in your vehicle

☐ Hearing about older driver safety or unsafe older drivers stories

☐ Near miss

☐ New diagnosis

☐ Health Issue

☐ Other (please describe):

33. *How easy do you believe it would be for you to meet your transportation needs if you were no longer driving yourself?*

Not Easy at All ☐ ☐ ☐ ☐ ☐ **Very Easy**

34. *How long do you expect to continue driving?*_____ years

35. *Have you driven in the last 30 days?*

☐ Yes ☐ No

↓

<u>IF NO</u>, how many years has it been since the last time you drove?
(Please write "0" if you drove in the last year.) _____ years

Next, please tell us more about your current and future transportation.

**36.** *How confident are you in your current driving skills and abilities?*	☐ ☐ ☐ ☐ ☐ **Not at All Confident** … **Very Confident**
**37.** *How confident are you that you could meet your transportation needs <u>if you were no longer driving yourself</u>?*	☐ ☐ ☐ ☐ ☐ **Not at All Confident** … **Very Confident**

**38.** *How much have you thought about a possible future time <u>when you are still driving, but drive less than you currently do?</u>*	☐ ☐ ☐ ☐ ☐ **Not at All** … **A Lot**
**39.** *How much have you thought about a possible future time when you are <u>no longer driving at all</u>?*	☐ ☐ ☐ ☐ ☐ **Not at All** … **A Lot**
**40.** *How much have you planned for a time in the future when you may no longer be driving?*	☐ ☐ ☐ ☐ ☐ **Not at All** … **A Lot**

41. *If you were no longer driving yourself, how well could <u>your future transportation needs be met</u> using each the following transportation methods?*

a. *RIDES WITH OTHER DRIVERS* (family, friends, etc.)	☐ ☐ ☐ ☐ ☐ **Not Well at All** … **Very Well**
b. *BUSES*	☐ ☐ ☐ ☐ ☐ **Not Well at All** … **Very Well**
c. *MASS TRANSPORT* (light rail, trains, etc.)	☐ ☐ ☐ ☐ ☐ **Not Well at All** … **Very Well**
d. *TAXIS/CABS*	☐ ☐ ☐ ☐ ☐ **Not Well at All** … **Very Well**
e. *SPECIALIZED TRANSPORT* (medical transport, disabled/senior shuttles, etc.)	☐ ☐ ☐ ☐ ☐ **Not Well at All** … **Very Well**
f. *WALKING* (for transportation, <u>NOT</u> for enjoyment or exercise exclusively)	☐ ☐ ☐ ☐ ☐ **Not Well at All** … **Very Well**
g. *"E-HAIL" APPS* (such as Uber or Lyft) on a smartphone or tablet	☐ ☐ ☐ ☐ ☐ **Not Well at All** … **Very Well**
h. OTHER<br>Please specify:______________________	☐ ☐ ☐ ☐ ☐ **Not Well at All** … **Very Well**

42. *How __comfortable__ would you be using each of the following transportation methods in the future if you were no longer driving?*

a. *RIDES WITH OTHER DRIVERS* (family, friends, etc.)	☐ ☐ ☐ ☐ ☐<br>**Not at All Comfortable** ... **Completely Comfortable**
b. *BUSES*	☐ ☐ ☐ ☐ ☐<br>**Not at All Comfortable** ... **Completely Comfortable**
c. *MASS TRANSPORT* (light rail, trains, etc.)	☐ ☐ ☐ ☐ ☐<br>**Not at All Comfortable** ... **Completely Comfortable**
d. *TAXIS/CABS*	☐ ☐ ☐ ☐ ☐<br>**Not at All Comfortable** ... **Completely Comfortable**
e. *SPECIALIZED TRANSPORT* (medical transport, disabled/senior shuttles, etc.)	☐ ☐ ☐ ☐ ☐<br>**Not at All Comfortable** ... **Completely Comfortable**
f. *WALKING* (for transportation, __NOT__ for enjoyment or exercise exclusively)	☐ ☐ ☐ ☐ ☐<br>**Not at All Comfortable** ... **Completely Comfortable**
g. *"E-HAIL" APPS* (such as Uber or Lyft) on a smartphone or tablet	☐ ☐ ☐ ☐ ☐<br>**Not at All Comfortable** ... **Completely Comfortable**
h. OTHER<br>Please specify:______________________	☐ ☐ ☐ ☐ ☐<br>**Not at All Comfortable** ... **Completely Comfortable**

43. *How likely would you be to use each of the following transportation methods if you were not driving in the future?*

a. *RIDES WITH OTHER DRIVERS* (family, friends, etc.)	☐ ☐ ☐ ☐ ☐ **Not at All Likely** … **Very Likely**
b. *BUSES*	☐ ☐ ☐ ☐ ☐ **Not at All Likely** … **Very Likely**
c. *MASS TRANSPORT* (light rail, trains, etc.)	☐ ☐ ☐ ☐ ☐ **Not at All Likely** … **Very Likely**
d. *TAXIS/CABS*	☐ ☐ ☐ ☐ ☐ **Not at All Likely** … **Very Likely**
e. *SPECIALIZED TRANSPORT* (medical transport, disabled/senior shuttles, etc.)	☐ ☐ ☐ ☐ ☐ **Not at All Likely** … **Very Likely**
f. *WALKING* (for transportation, NOT for enjoyment or exercise exclusively)	☐ ☐ ☐ ☐ ☐ **Not at All Likely** … **Very Likely**
g. *"E-HAIL" APPS* (such as Uber or Lyft) on a smartphone or tablet	☐ ☐ ☐ ☐ ☐ **Not at All Likely** … **Very Likely**
h. OTHER<br>Please specify:______________________	☐ ☐ ☐ ☐ ☐ **Not at All Likely** … **Very Likely**

In addition to what you are doing, we are interested in learning how much have you talked to other people about a time when you are no longer driving.

	a. *How much have you discussed a possible nondriving future with this person or people?*	b. *Do you think they want you to plan MORE for a nondriving future?*	c. *How much do you care about if they want you to plan more?*
**44.** Spouse/ Partner	☐ Have not talked<br>☐ Talked in passing<br>☐ Seriously talked<br>☐ Do not have a spouse/partner<br>(*If not, please move on to question 45.*)	☐ Not at all<br>☐ A little<br>☐ Some<br>☐ A lot	☐ Not at all<br>☐ A little<br>☐ Some<br>☐ A lot
**45.** Adult Children/ Grandchildren	☐ Have not talked<br>☐ Talked in passing<br>☐ Seriously talked<br>☐ Do not have adult children/grandchildren<br>(*If not, please move on to question 46.*)	☐ Not at all<br>☐ A little<br>☐ Some<br>☐ A lot	☐ Not at all<br>☐ A little<br>☐ Some<br>☐ A lot
**46.** Healthcare Providers (including primary care physicians, eye doctors, nurses, etc.)	☐ Have not talked<br>☐ Talked in passing<br>☐ Seriously talked<br>☐ Do not have healthcare providers<br>(*If not, please move on to question 47.*)	☐ Not at all<br>☐ A little<br>☐ Some<br>☐ A lot	☐ Not at all<br>☐ A little<br>☐ Some<br>☐ A lot
**47.** Others Please specify:	☐ Have not talked<br>☐ Talked in passing<br>☐ Seriously talked	☐ Not at all<br>☐ A little<br>☐ Some<br>☐ A lot	☐ Not at all<br>☐ A little<br>☐ Some<br>☐ A lot

If you are skipping forward from page 2 or 4, please start again here.

Consider what would happen if you could not get yourself to valued destinations and activities independently. Maybe this is occurring already in your life; maybe it could happen in the future.

48. Read each statement and consider if you agree or disagree and how strongly. Mark your answer by circling the appropriate number to the right. Respond to all items if possible.

	**Strongly DISAGREE**				**Strongly AGREE**
a. *Mobility loss can be sudden or progressive, but it is always devastating.*	**1**	**2**	**3**	**4**	**5**
b. *Asking others for help with mobility means that I am losing my independence.*	**1**	**2**	**3**	**4**	**5**
c. *I am a burden if I ask others for help with transportation.*	**1**	**2**	**3**	**4**	**5**
d. *I avoid thinking about losing my mobility.*	**1**	**2**	**3**	**4**	**5**
e. *I wish others would stop talking to me about my mobility.*	**1**	**2**	**3**	**4**	**5**
f. *Asking for a ride creates an inconvenience for others.*	**1**	**2**	**3**	**4**	**5**
g. *Other people simply don't understand what it's like to have limited mobility.*	**1**	**2**	**3**	**4**	**5**
h. *It is devastating for older people to have someone take away their car keys.*	**1**	**2**	**3**	**4**	**5**
i. *I do not like to ask others for a ride.*	**1**	**2**	**3**	**4**	**5**
j. *I feel depressed at the thought of being limited in my mobility.*	**1**	**2**	**3**	**4**	**5**

	Strongly <u>DIS</u>AGREE				Strongly AGREE
k. *Moving to a retirement community is too restrictive for my desired mobility.*	**1**	**2**	**3**	**4**	**5**
l. *When I see older people with significant limitations in mobility, I fear that I will end up like that too.*	**1**	**2**	**3**	**4**	**5**
m. *There is no way to plan for loss of mobility in aging.*	**1**	**2**	**3**	**4**	**5**
n. *A big loss of mobility would really hurt my self-esteem.*	**1**	**2**	**3**	**4**	**5**
o. *Loss of mobility is very isolating and depressing.*	**1**	**2**	**3**	**4**	**5**
p. *I shudder to think of a time when I am less mobile than I am now.*	**1**	**2**	**3**	**4**	**5**
q. *I refuse to accept that I might lose my mobility in the future.*	**1**	**2**	**3**	**4**	**5**
r. *My future independence hinges on my ability to get myself around.*	**1**	**2**	**3**	**4**	**5**
s. *I have not thought much about my future mobility before today.*	**1**	**2**	**3**	**4**	**5**
t. *I've seen others become frail and immobile in older age, and I am determined to avoid this fate at whatever cost.*	**1**	**2**	**3**	**4**	**5**
u. *It really frustrates me when I have difficulty getting around.*	**1**	**2**	**3**	**4**	**5**
v. *I feel angry when I think about losing my mobility.*	**1**	**2**	**3**	**4**	**5**
w. *I feel self-conscious when my mobility needs become a concern for others.*	**1**	**2**	**3**	**4**	**5**
x. *It is not easy for me to ask for help with transportation when I need it.*	**1**	**2**	**3**	**4**	**5**

Please share how much you have planned for the following future needs. Mark the appropriate box for each topic below.

How much have you planned for your possible future...

**49.** *...general health care needs?*	☐ ☐ ☐ ☐ ☐<br>**Not at All** **A Lot**
**50.** *...financial matters?*	☐ ☐ ☐ ☐ ☐<br>**Not at All** **A Lot**
**51.** *...housing or living arrangements?*	☐ ☐ ☐ ☐ ☐<br>**Not at All** **A Lot**
**52.** *...personal healthcare?*	☐ ☐ ☐ ☐ ☐<br>**Not at All** **A Lot**
**53.** *...end-of-life decisions?*	☐ ☐ ☐ ☐ ☐<br>**Not at All** **A Lot**
**54.** *...estate planning and/or will?*	☐ ☐ ☐ ☐ ☐<br>**Not at All** **A Lot**

Finally, we'd like to know some more general information about you.

55. In general, would you say your health is:

- ☐ Excellent
- ☐ Very Good
- ☐ Good
- ☐ Fair
- ☐ Poor

The following two questions are about activities you might do during a typical day. Does YOUR HEALTH NOW LIMIT YOU in these activities? If so, how much?

56. *MODERATE ACTIVITIES, such as moving a table, pushing a vacuum cleaner, bowling, or playing golf:*

- ☐ Yes, Limited A Lot
- ☐ Yes, Limited A Little
- ☐ No, Not Limited At All

57. *Climbing SEVERAL flights of stairs:*

- ☐ Yes, Limited A Lot
- ☐ Yes, Limited A Little
- ☐ No, Not Limited At All

During the PAST 4 WEEKS have you had any of the following problems with your work or other regular activities AS A RESULT OF YOUR PHYSICAL HEALTH?

58. *ACCOMPLISHED LESS than you would like:* ☐ Yes ☐ No

59. *Were limited in the KIND of work or other activities:* ☐ Yes ☐ No

During the PAST 4 WEEKS, were you limited in the kind of work you do or other regular activities AS A RESULT OF ANY EMOTIONAL PROBLEMS (such as feeling depressed or anxious)?

60. *ACCOMPLISHED LESS than you would like:* ☐ Yes ☐ No

61. *Didn't do work or other activities as CAREFULLY as usual:* ☐ Yes ☐ No

62. *During the PAST 4 WEEKS, how much did PAIN interfere with your normal work (including both work outside the home and housework)?*

- ☐ Not At All
- ☐ A Little Bit
- ☐ Moderately
- ☐ Quite A Bit
- ☐ Extremely

The next three questions are about how you feel and how things have been DURING THE PAST 4 WEEKS. For each question, please give the one answer that comes closest to the way you have been feeling. How much of the time during the PAST 4 WEEKS –

63. *Have you felt calm and peaceful?*

- ☐ All of the Time
- ☐ Most of the Time
- ☐ A Good Bit of the Time
- ☐ Some of the Time
- ☐ A Little of the Time
- ☐ None of the Time

64. *Did you have a lot of energy?*

- ☐ All of the Time
- ☐ Most of the Time
- ☐ A Good Bit of the Time
- ☐ Some of the Time
- ☐ A Little of the Time
- ☐ None of the Time

65. *Have you felt downhearted and blue?*

- ☐ All of the Time
- ☐ Most of the Time
- ☐ A Good Bit of the Time
- ☐ Some of the Time
- ☐ A Little of the Time
- ☐ None of the Time

66. *During the PAST 4 WEEKS, how much of the time has your PHYSICAL HEALTH OR EMOTIONAL PROBLEMS interfered with your social activities (like visiting with friends, relatives, etc.)?*

- ☐ All of the Time
- ☐ Most of the Time
- ☐ A Good Bit of the Time
- ☐ Some of the Time
- ☐ A Little of the Time
- ☐ None of the Time

67. *What is your current age?* _____ years old

68. *To what age do you expect to live?* _____ years old

69. *What is the highest grade of school or year of college you completed?*

- ☐ Less than high school

  *IF LESS THAN HIGH SCHOOL, what was the last grade you finished?*____
- ☐ High school diploma
- ☐ Some college
- ☐ College graduate
- ☐ Some graduate/professional school
- ☐ Master's/Professional degree
- ☐ Doctorate

70. *What is your gender?* ___________________________

71. *What race do you consider yourself to be? Please mark all that apply.*

☐ White/Caucasian ☐ Black/African-American

☐ Other (Please specify):___________________________

72. *Do you consider yourself Hispanic or Latino?* ☐ Yes ☐ No ☐ Not sure

73. *How would you describe the area where you live?*

- ☐ Urban (City) ☐ Rural ☐ Suburban

74. *How would you describe your current employment status?*

- ☐ Working full-time
- ☐ Working part-time
- ☐ Temporarily laid off
- ☐ Unemployed and looking for work
- ☐ Disabled and unable to work
- ☐ Retired
- ☐ Homemaker
- ☐ Other (please describe):__________________________

75. *Which best describes your yearly household income?*

- ☐ Less than $10,000
- ☐ $10,000 to $14,999
- ☐ $15,000 to $24,999
- ☐ $25,000 to $49,999
- ☐ $50,000 to $99,999
- ☐ $100,000 to $149,999
- ☐ $150,000 to $199,999
- ☐ $200,000 and above

76. *What is your current relationship status?*

- ☐ Single (never married)
- ☐ Married/Domestic partnership
- ☐ Divorced/Separated
- ☐ Widowed

Thank you for completing the survey! Please mail it back in the envelope included in the package. You should get your $20 gift card within 3-4 weeks after we receive your survey.
